# Sex effects on DNA methylation affect discovery in epigenome-wide association study of schizophrenia

**DOI:** 10.1038/s41380-024-02513-9

**Published:** 2024-03-19

**Authors:** Markos Tesfaye, Leticia M. Spindola, Anne-Kristin Stavrum, Alexey Shadrin, Ingrid Melle, Ole A. Andreassen, Stephanie Le Hellard

**Affiliations:** 1 NORMENT, Division of Mental Health and Addiction, Oslo University Hospital and Institute of Clinical Medicine, University of Oslo, Oslo, Norway; 2 NORMENT, Department of Clinical Science, University of Bergen, Bergen, Norway; 3 Dr. Einar Martens Research Group for Biological Psychiatry, Department of Medical Genetics, Haukeland University Hospital, Bergen, Norway; 4 Bergen Center for Brain Plasticity, Haukeland University Hospital, Bergen, Norway; 5 KG Jebsen Centre for Neurodevelopmental Disorders, University of Oslo and Oslo University Hospital, Oslo, Norway

**Keywords:** Genetics, Biological techniques

## Abstract

Sex differences in the epidemiology and clinical characteristics of schizophrenia are well-known; however, the molecular mechanisms underlying these differences remain unclear. Further, the potential advantages of sex-stratified meta-analyses of epigenome-wide association studies (EWAS) of schizophrenia have not been investigated. Here, we performed sex-stratified EWAS meta-analyses to investigate whether sex stratification improves discovery, and to identify differentially methylated regions (DMRs) in schizophrenia. Peripheral blood-derived DNA methylation data from 1519 cases of schizophrenia (male *n* = 989, female *n* = 530) and 1723 controls (male *n* = 997, female *n* = 726) from three publicly available datasets, and the TOP cohort were meta-analyzed to compare sex-specific, sex-stratified, and sex-adjusted EWAS. The predictive power of each model was assessed by polymethylation score (PMS). The number of schizophrenia-associated differentially methylated positions identified was higher for the sex-stratified model than for the sex-adjusted one. We identified 20 schizophrenia-associated DMRs in the sex-stratified analysis. PMS from sex-stratified analysis outperformed that from sex-adjusted analysis in predicting schizophrenia. Notably, PMSs from the sex-stratified and female-only analyses, but not those from sex-adjusted or the male-only analyses, significantly predicted schizophrenia in males. The findings suggest that sex-stratified EWAS meta-analyses improve the identification of schizophrenia-associated epigenetic changes and highlight an interaction between sex and schizophrenia status on DNA methylation. Sex-specific DNA methylation may have potential implications for precision psychiatry and the development of stratified treatments for schizophrenia.

## Introduction

Schizophrenia is a severe psychiatric disorder characterized by signs and symptoms related to anomalies in thoughts, perception, and behavior [1]. Schizophrenia exhibits differences between males and females in the age of onset and risk of negative versus affective symptoms. Abnormalities in neurodevelopmental processes during the prenatal period probably play a role in the pathophysiology of schizophrenia [2, 3]. These processes are to a large extent under the control of genetic and gene expression regulatory mechanisms [4]. DNA methylation, the addition of a methyl group to the cytosine of CpG, is a mechanism for gene regulation. This process is age and sex dependent and can mediate the influence of genetic and environmental factors on neurodevelopment [2, 5]. In the fetal brain, dynamic changes in DNA methylation overlap with the periods of shifts in predominant neurodevelopmental processes [3, 6]. Furthermore, DNA methylation loci associated with fetal neurodevelopmental stages were reported to be enriched for genes associated with the risk of developing schizophrenia [2].

Males and females exhibit widespread differences in DNA methylation across different tissues (e.g., brain, blood, buccal mucosa) [7–9]. These differences involve both autosomal and sex chromosomes [9]. In the fetal brain, the divergence of DNA methylation between males and females appears at the beginning of the second trimester coinciding with the surge in fetal gonadal steroids [6]. A causal link between sex differences in fetal brain DNA methylation and estradiol has been demonstrated in animal models [10]. A lifelong pattern of global hypermethylation in females relative to males is reported in a wide range of human tissues including the brain [9, 11–14]. DNA methylation loci exhibiting sex differences are reported to show enrichment for genes associated with psychiatric disorders (e.g., schizophrenia) [7, 13, 15], and molecular pathways related to nervous system development [9, 15]. Also, sex differential methylation has been linked to sex differential transcriptomics in the prefrontal cortex [16], and may underlie the male-female differences in schizophrenia [15, 17].

Sex differences in DNA methylation loci associated with schizophrenia are reported in the brain with a higher extent of epigenetic dysregulation in females [17]. In genome-wide association studies (GWAS), it was demonstrated that sex-adjusted models combining both sexes are not optimal for identifying loci exhibiting heterogeneous allelic effects between males and females [18]. Therefore, standard sex-adjusted models offer a limited power to discover loci with sex effects and may hinder progress in identifying molecular pathways linked to a phenotype. The authors have suggested sex-stratified meta-analysis whereby male-only and female-only GWASs are combined in meta-analyses to obtain substantial gain in power [18]. Considering the reported sex differences in DNA methylation level which may be more pronounced than in genetics, sex-stratified meta-analyses may boost power leading to novel discoveries in epigenome-wide association studies (EWASs) [8]. However, no published EWAS studies to date have systematically investigated the potential advantages of sex-stratified over the common sex-adjusted analysis.

Here, we conducted meta-analyses of four EWASs of schizophrenia and presented a systematic comparison of sex-stratified and sex-adjusted approaches. We hypothesized that the former improves epigenomic discoveries in schizophrenia. We also performed male-only and female-only meta-analyses to elucidate common and sex-specific biological mechanisms in schizophrenia. Finally, we compared the performance of polymethylation scores (PMS) computed from the different meta-analyses in predicting schizophrenia in an independent sample.

## Materials and methods

### Cohort characteristics

#### Thematically Organized Psychosis (TOP) Sample

The TOP study is the main observational clinical research protocol being conducted by the Norwegian Center for Mental Disorders Research (NORMENT, Norway). The study was approved by the Regional Committees for Medical Research Ethics South-East Norway and the Norwegian Data Inspectorate (REK # 2009/2485). All participants provided written informed consent. Four hundred sixty-two individuals with schizophrenia were recruited from inpatient and outpatient psychiatric facilities in Norway. Diagnoses were ascertained using the Structured Clinical Interview for DSM-IV Axis 1 disorder (SCID-I). Healthy controls comprised 765 adults living in the greater Oslo area who were randomly selected using the Norwegian National Population Registry. All study participants were of European ancestry. Detailed information on the cohort is provided elsewhere [19].

#### Publicly available DNA methylation datasets

We obtained DNA methylation datasets of three cohorts of individuals with schizophrenia and healthy controls from the Gene Expression Omnibus, a public functional genomics data repository (https://www.ncbi.nlm.nih.gov/geo/). We briefly describe the dataset below and more detailed information is published elsewhere [20].

##### University College London dataset (UCL, GSE80417)

The UCL dataset comprised normalized betas of DNA methylation from 353 individuals with schizophrenia and 322 controls recruited from the United Kingdom (UK). An ICD-10 clinical diagnosis of schizophrenia was further ascertained through interviews using the Schedule for Affective Disorders and Schizophrenia-Lifetime Version. Controls included individuals from the UK who did not have a personal history of mental disorder, or a family history of schizophrenia, bipolar disorder, and alcohol dependence [21].

##### Aberdeen dataset (ABR, GSE84727)

The ABR dataset was also normalized betas of DNA methylation obtained from 414 individuals with schizophrenia and 433 controls predominantly from Scotland (95%). A DSM-IV or ICD-10 diagnosis of schizophrenia was validated by using the Operational Criteria Checklist (OPCRIT). Controls were volunteers from general practices excluding individuals with a history of major mental illness in themselves or their first-degree relatives. More detailed information on the characteristics of study participants is published elsewhere [20, 22].

##### Institute of Psychiatry, Psychology, and Neuroscience dataset (IoPPN, GSE152027)

The processed DNA methylation beta value dataset was obtained for the 290 individuals with schizophrenia and 203 controls. The study participants were recruited through the South London and Maudsley Mental Health National Health Service (NHS) Foundation Trust. Clinical diagnoses were ascertained by administering the Schedules for Clinical Assessment in Neuropsychiatry (SCAN). Participants were of White, African, and Asian ancestries. Individuals with psychoses due to underlying medical conditions were excluded. Controls were volunteers recruited from the local population living within the same NHS catchment area as the cases. Controls who had a previous diagnosis of psychotic illness or who met the criteria for psychotic disorder on the Psychosis Screening Questionnaire were excluded. More detailed information on the recruitment of study participants is provided in previous publications [23, 24].

### DNA methylation

For each cohort, peripheral whole blood was collected from the participants and DNA was extracted following the standard protocol. DNA methylation was assessed with the Illumina Infinium HumanMethylation450 BeadChip (ABR, IoPPN, UCL), or Infinium MethylationEPIC (TOP) arrays (Illumina, San Diego, CA). The cohorts performed sample processing, quality control (QC), and normalization based on the protocols described [20, 25]. As part of the QC, samples with >1% of probes with detection *p*-value > 0.05 and probes with >1% of samples with detection *p*-value > 0.05 were removed from ABR, IoPPN, and UCL data. In the TOP data, a slightly more stringent procedure was followed with the removal of probes with a detection *p*-value >  0.01 in at least 1% of the samples and the removal of samples where at least 1% of the probes had a detection *p*-value >  0.01 using *wateRmelon* package [26]. Finally, the preprocessed, normalized, beta values ranging from 0 (unmethylated) to 1 (fully methylated) were used for the EWAS.

### Covariates

Models were adjusted for the following potential confounders: age, smoking scores, estimated cell-type proportions, batch effect, genotype principal components (PCs), methylation PCs, and surrogate variables. For the sex-specific and sex-stratified analyses, adjustment for covariates was done separately for the male and female data. Sex was included as a covariate only in sex-adjusted models. Missing age data were imputed using the online tool DNA methylation age calculator (https://dnamage.genetics.ucla.edu/) [27]. Smoking scores were computed from the DNA methylation data using the methods published by Zeilinger et al. [28], and Elliot et al. [29]. Similarly, estimated cell-type proportions were computed using the *projectCellType_CP* function of the FlowSorted.Blood.450k package (for ABR, IoPPN, UCL cohorts) [30], and using the *estimateCellCounts2* function of FlowSorted.Blood.EPIC package for the TOP cohort [31].

### Statistical analyses

#### Epigenome-wide association analyses (EWAS)

For each cohort, linear regression analysis was performed to identify differentially methylated probes (DMPs) associated with schizophrenia using *limma* package [32]. DNA methylation beta values for each probe were regressed against schizophrenia case-control status along with covariates stated above. For the TOP sample, ten genotype principal components were included in the model. A similar approach was followed for the male-only and female-only analyses except for the exclusion of sex from covariates. Additionally, linear regression models were run to examine the interaction between sex and schizophrenia status on DNA methylation. Probes mapped on the sex chromosomes (X and Y) and probes used to derive covariates such as missing ages, cell proportions and smoking scores were excluded from the analyses.

#### Meta-analyses

Four different meta-analyses were performed: sex-adjusted (i.e. sex is included as a covariate in the regression), male-only, female-only, and sex-stratified. Each meta-analysis involved four cohorts except the sex-stratified meta-analysis which involved eight cohorts. The differences in the number of cohorts in the meta-analyses is accounted for by the degrees of freedom. The log fold change (logFCs) and standard error (SEs) from each EWAS were corrected using *bacon* R package which applies a Bayesian approach to control false positive findings [33]. All meta-analyses were performed using corrected logFCs and SEs in the *metagen* function of the *meta* R package. The *metagen* function (https://cran.r-project.org/web/packages/meta/index.html) implements standard inverse variance meta-analysis where the pooled effect sizes are computed as the weighted average of effect sizes in meta-analyzed EWAS. Specifically, for a given CpG site the weight is defined as the inverse variance of its effect size estimate which reflects the heterogeneity of the phenotype across measured methylation levels (Supplementary Information Fig. S1).

Probes with results from EWAS in at least two cohorts were included in the meta-analyses. Effect sizes for random effects model and two-sided tests for the *p*-values were computed. The effect estimates of the interaction between sex and schizophrenia on DNA methylation were meta-analyzed using the same methods. We present the number of DMPs identified using two *p*-value cut-offs, 2.4 × 10^−^^7^ and 3.6 × 10^−^^8^, corresponding to the 450 K and genome-wide significance thresholds as recommended previously [34].

Differentially methylated regions (DMRs) were identified by applying the *comb-p* procedure [35], specifying parameters as seed *p*-value  = 0.001 and maximum distance between probes of 750 base pairs [36]. DMRs with at least four probes were identified at a significance threshold of *p* < 0.05 after Sidak correction for multiple testing [36].

#### Simulation of sex-stratified and sex-adjusted meta-analyses

We set up a simulation of 400 individuals (50% males) and a total of 10,000 CpGs with normally distributed ‘methylation’ measurements. One hundred randomly selected CpGs had non-zero effect sizes drawn from a normal distribution, effect sizes of the remaining 9900 CpGs were set to zero. The average non-zero effect size in males was fixed at 0.1 while the average non-zero effect sizes in females were different in four different scenarios (i.e., 0.1, 0.2, 0.4, and 0.8). The synthetic normally distributed phenotype for each individual was calculated as a sum of ‘methylation’ measurements multiplied by their effect sizes and the normally distributed effect of ‘non-methylation’ factors, where the latter explains 50% of the total phenotypic variability. Finally, sex-adjusted analyses (in which sexes were analyzed together) where sex is included as a covariate were compared with sex-stratified fixed-effect meta-analyses. The resulting QQ plots were visually examined for upward and leftward deviations that indicate a boost in statistical power.

#### Polymethylation scores

We calculated polymethylation scores (PMS) to evaluate the performance of the four meta-analyses (sex-stratified, sex-adjusted, male-only, female-only) on an independent sample. The summary statistics obtained from meta-analyses of the ABR, UCL, and IoPPN cohorts (i.e., thus excluding the TOP cohort), served as the training datasets and the TOP cohort served as the target (test) dataset.

The PMSs were computed following similar approaches to the polygenic risk score calculation in genetics [37]. To account for correlations among DNA methylation at proximal CpG sites, we applied CoMeBack (Co-Methylation with genomic CpG Background) on the DNA methylation data of the target dataset [38, 39]. CoMeBack employs a sliding window approach to estimate DNA co-methylation. Briefly, it connects adjacent array probes and assesses the correlations between DNA methylation levels for all connected probes [38, 39]. If all pairs of adjacent probes within a unit exhibit a correlation square (R^2^) greater than 0.3, that unit is identified as a co-methylated region. A “pruning” step was then performed, keeping only the CpG site with the most significant *p*-value in the meta-analysis summary statistics. The CoMeBack step was performed using the pipeline made available by Chen et al. [38]. We used the EWAS residualized DNA methylation betas to estimate DNA co-methylation in the TOP cohort.

Following the CoMeBack step, we calculated multiple PMS across *p*-value thresholds and used a method described for PRS to avoid thresholding optimization [37]. This involves performing principal component analysis (PCA) on the set of PMS and using the first principal component as the PMS for association testing with the schizophrenia case-control status. The number of *p*-value thresholds used in the PCA was standardized across the different PMS calculations. The *p*-value thresholds were: 0.5, 5.0 × 10e-02, 5.0 × 10e-03, 5.0 × 10e-04, 5.0 × 10e-05, and 5.0 × 10e-06.

The performance of the three training datasets was evaluated in the target (test) datasets of TOP total (*n* = 1227), TOP male (*n* = 682), and TOP female (*n* = 545) samples. The variance in schizophrenia liability (Nagelkerke R^2^) explained by each PMS was estimated using logistic regression models. To assess whether there were statistically significant differences in the prediction of schizophrenia between models based on the different training datasets, we performed a non-nested hypothesis test and calculated the Akaike Information Criterion (AIC) and the Bayesian Information Criterion (BIC) model fit metrics.

#### Functional annotations and regulatory enrichment

The potential effect of genetic variants on the DNA methylation levels of the DMPs associated with schizophrenia was examined. This was performed by searching the identified DMPs in the list of known methylation quantitative trait loci (meQTLs) in the human blood at different stages of life (at birth, childhood, adolescence, middle age, and during pregnancy) [40].

We used IlluminaHumanMethylation450kanno.ilmn12.hg19 Bioconductor package [41] to annotate the DMPs and DMRs identified. When this approach did not yield annotations, the specific CpGs were examined in the UCSC genome browser (https://genome-euro.ucsc.edu/; human genome build 19) to annotate whether they were located in an alternative transcript of a reference sequence gene or were annotated to the nearest gene (ng) within 50 kb [42]. The genes annotated to the DMRs associated with schizophrenia were then analyzed for enrichment of biological processes, and cellular components using the functional mapping and gene annotation (FUMA) platform [43].

## Results

### Cohort characteristics

Our study comprised four cohorts with a total of 1519 individuals with schizophrenia and 1723 controls. Overall, there were more males in both cases (65%) and control groups (58%), and cases were older, 41.6( ± 14.2) years than controls 36.7( ± 12.4) years. Smoking scores and cell proportions also differed between cases and controls (Table 1). Individual level data on treatments are not available for cases of schizophrenia, however, the majority (70 to 100%) had received antipsychotic medications [20].

**Table 1:** Characteristics of cohort participants.

	Cases					Controls				
	ABR	IoPPN	TOP	UCL	Total	ABR	IoPPN	TOP	UCL	Total
Sample size	414	290	462	353	1519	433	203	765	322	1723
Sex^a^										
* Male*	283	193	259	254	989	319	113	423	142	997**
* Female*	131	97	203	99	530	114	90	342	180	726
Age^b^										
* Mean (SD)*	47.9 (14.0)	46.6 (9.6)	31.3 (10.4)	43.8 (14.5)	41.6 (14.2)	45.2 (12.2)	30.8 (10.4)	33.2 (8.7)	37.6 (14.9)	36.7 (12.4)**
Smoking scores^b^										
* Mean (SD)*	7.4 (6.4)	8.5 (6.4)	−3.7 (4.4)	6.7 (6.1)	4.0 (7.8)	2.6 (5.3)	2.4 (4.2)	−5.4 (3.0)	0.2 (4.7)	−1.4 (5.5)**
Cell proportion (%)^a^										
* Monocytes*	7.3	8.6	7.5	8.5	7.9	7.3	8.1	7.2	8.6	7.6**
* Granulocytes*	55.8	49.6	53.7	54.2	53.6	51.7	49.6	51.2	51.0	51.1**
* Natural killer cells*	4.8	5.8	6.6	6.4	5.9	5.0	5.8	7.3	6.7	6.5**
* CD4* + *T-cells*	15.5	17.0	20.4	18.5	18.0	17.4	16.6	20.3	18.7	18.8**
* CD8* + *T-cells*	4.8	6.6	12.2	3.6	7.1	6.6	8.4	13.0	5.9	9.5**
* B-cells*	4.5	5.8	7.1	5.0	5.7	4.4	5.2	6.8	5.0	5.7

*ABR* Aberdeen, *IoPPN* Institute of Psychiatry, Psychology, and Neuroscience, *TOP* Thematically Organized Psychosis, *UCL* University College London, *SD* standard deviation

^a^Chi-square test for comparison of proportions between cases and controls.

^b^T-tests for comparison of means between cases and controls.

**P < 0.001.

### Sex-specific, sex-stratified, and sex-adjusted DNA methylation differences in schizophrenia

**Fig. 1:**
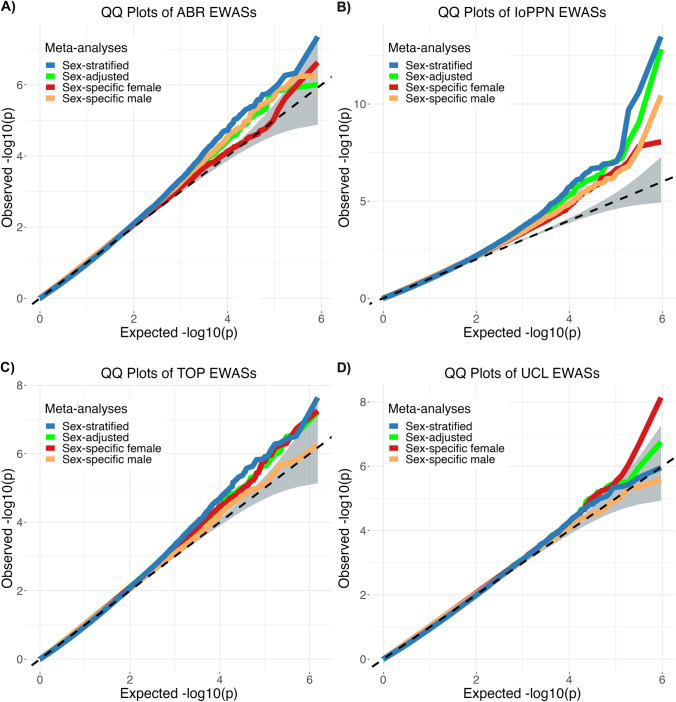
Quantile-Quantile (QQ) plots of *p*-values from sex-specific, sex-adjusted, and sex-stratified genome-wide DNA methylation analyses in schizophrenia for four cohorts. **A** Aberdeen (ABR), **B** Institute of Psychiatry, Psychology and Neuroscience (IoPPN), **C** Thematically Organized Psychosis (TOP), and **D** University College London (UCL). EWAS epigenome-wide association study.

For each cohort, we performed three EWAS, sex-specific (female-only, male-only) and sex-adjusted (total sample). We then performed four meta-analyses: sex-specific female (cases = 530 and controls = 726), sex-specific male (cases = 989 and controls = 997), sex-stratified female + male (total sample), and sex-adjusted (total sample). Cohort-specific and sex-specific EWAS *p*-values exhibited an acceptable degree of inflation after BACON correction (Fig. 1).

In males, we found hypermethylation at cg11269166 (*METTL8*), and in females hypomethylation at cg18096987 (*VGLL4*) and cg04276536 (*CCDC102* *A*), and hypermethylation at cg11854073 (*SLC9A10*) to be associated with schizophrenia (Table 2; Supplementary Information Figs. S2 & S3). Using random effects model, the interaction between sex and schizophrenia status on DNA methylation were significant for cg11854073 and cg04276536. Our results also found that the sex interaction effects were marginally significant for cg11269166 (*P* < 0.51) and non-significant for cg18096987 (Supplementary Table S1).

**Table 2:** Comparison of random effects of schizophrenia-associated DNA methylation sites in sex-specific, sex-stratified, and sex-adjusted meta-analyses.

CpG	CHR	Position (Human genome build 19)	Gene^a^	Female sample^b^		Male sample^b^		Sex-adjusted meta-analysis^b^		Sex-stratified meta-analysis^b^	
				Effect size ± S.E.	*P*-value	Effect size ± S.E.	*P*-value	Effect size ± S.E.	*P*-value	Effect size ± S.E.	*P*-value
cg02488934	1	156422580	*MEF2D*^c^	0.66 ± 0.16	3.18E-05	0.67 ± 0.14	1.95E-06	0.54 ± 0.10	**1.33E-07**	0.67 ± 0.11	**2.61E-10**
cg27541604^d^	1	159046451	*AIM2*	0.74 ± 0.18	2.51E-05	0.73 ± 0.25	3.33E-03	0.68 ± 0.17	8.83E-05	0.71 ± 0.12	**7.72E-10**
cg02934509	1	1669413	*SLC35E2*	0.92 ± 0.19	1.86E-06	0.54 ± 0.16	8.51E-04	0.66 ± 0.12	**1.28E-07**	0.70 ± 0.12	**1.88E-08**
cg07600998	1	147146108	*ACP6*^c^	0.56 ± 0.12	3.55E-06	0.40 ± 0.15	6.44E-03	0.45 ± 0.11	3.55E-05	0.47 ± 0.09	**1.32E-07**
cg24935896	1	201431492	*PHLDA3*^c^	0.46 ± 0.17	8.15E-03	0.59 ± 0.14	1.90E-05	0.47 ± 0.10	5.44E-06	0.54 ± 0.10	**1.56E-07**
cg20994118	1	209780966	*CAMK1G*	0.72 ± 0.21	7.92E-04	0.68 ± 0.23	2.76E-03	0.58 ± 0.18	1.01E-03	0.69 ± 0.13	**2.40E-07**
cg11269166	2	172203847	*METTL8*	0.40 ± 0.25	1.01E-01	0.97 ± 0.19	**2.02E-07**	0.72 ± 0.22	1.23E-03	0.77 ± 0.20	9.67E-05
cg18096987	3	11623873	*VGLL4*	-0.98 ± 0.18	**7.91E-08**	-0.50 ± 0.15	8.10E-04	-0.55 ± 0.11	8.90E-07	-0.69 ± 0.12	**2.11E-09**
cg11854073	3	112013357	*SLC9A10*	2.80 ± 0.53	**1.33E-07**	1.29 ± 0.47	5.91E-03	1.84 ± 0.36	2.85E-07	2.00 ± 0.46	1.21E-05
cg25432300	5	90508265	*ADGRV1*^c^	0.90 ± 0.22	5.93E-05	0.69 ± 0.18	1.13E-04	0.75 ± 0.14	**5.98E-08**	0.77 ± 0.14	**3.33E-08**
cg15852826	6	31528915	*NFKBIL1*^c^	0.54 ± 0.16	6.09E-04	0.58 ± 0.13	6.78E-06	0.46 ± 0.10	3.97E-06	0.57 ± 0.10	**1.58E-08**
cg13675015	6	43217925	*TTBK1*	0.62 ± 0.16	1.45E-04	0.51 ± 0.14	3.50E-04	0.51 ± 0.11	2.25E-06	0.55 ± 0.11	**2.09E-07**
cg12269535	6	43142014	*SRF*	-0.98 ± 0.24	5.75E-05	-0.60 ± 0.20	2.77E-03	-0.83 ± 0.15	**5.02E-08**	-0.75 ± 0.15	1.12E-06
cg01973676	7	101596404	*CUX1*	1.09 ± 0.29	1.75E-04	0.61 ± 0.19	1.38E-03	0.67 ± 0.16	1.92E-05	0.79 ± 0.15	**7.38E-08**
cg24107852^e^	7	127960508	*RBM28*	-0.70 ± 0.25	4.28E-03	-0.81 ± 0.19	2.14E-05	-0.76 ± 0.20	1.42E-04	-0.76 ± 0.14	**1.05E-07**
cg19507068	7	79764176	*GNAI1*	0.59 ± 0.14	2.57E-05	0.44 ± 0.14	1.10E-03	0.42 ± 0.09	9.92E-06	0.52 ± 0.10	**1.36E-07**
cg26547058^d^	8	142243143	*SLC45A4*^f^	1.36 ± 0.34	5.51E-05	1.22 ± 0.37	8.68E-04	1.13 ± 0.30	1.55E-04	1.25 ± 0.21	**5.99E-09**
cg01142735	8	103932076	*MAILR*^f^	0.59 ± 0.16	3.14E-04	0.46 ± 0.12	2.29E-04	0.41 ± 0.10	4.01E-05	0.51 ± 0.10	**1.55E-07**
cg18919106^e^	8	132918665	*EFR3A*	-0.88 ± 0.28	2.11E-03	-0.53 ± 0.14	1.60E-04	-0.55 ± 0.16	5.40E-04	-0.65 ± 0.12	**1.64E-07**
cg04780563	10	35748463	*CCNY*	-1.17 ± 0.41	4.27E-03	-1.05 ± 0.29	2.90E-04	-1.10 ± 0.18	**6.96E-10**	-1.10 ± 0.20	**4.55E-08**
cg27207470^e^	11	111848326	*DIXDC1*	1.68 ± 0.38	9.77E-06	1.07 ± 0.32	7.41E-04	1.21 ± 0.25	8.53E-07	1.32 ± 0.24	**5.88E-08**
cg02419321	12	54811762	*ITGA5*	-0.48 ± 0.34	1.55E-01	-0.63 ± 0.14	4.53E-06	-0.45 ± 0.11	3.35E-05	-0.58 ± 0.11	**1.41E-07**
cg14327296	14	101051064	*BEGAIN*^f^	0.55 ± 0.20	7.31E-03	0.47 ± 0.14	6.63E-04	0.37 ± 0.09	1.67E-05	0.47 ± 0.09	**1.06E-07**
cg01678084^e^	15	67022087	*SMAD6*	0.68 ± 0.16	2.11E-05	0.45 ± 0.17	7.67E-03	0.45 ± 0.10	6.11E-06	0.53 ± 0.10	**1.52E-07**
cg06934654	16	89180742	*ACSF3*	0.49 ± 0.16	2.40E-03	0.39 ± 0.10	7.05E-05	0.38 ± 0.08	8.71E-07	0.42 ± 0.08	**2.90E-08**
cg06800849	16	89180587	*ACSF3*	0.70 ± 0.16	1.39E-05	0.46 ± 0.13	6.89E-04	0.50 ± 0.10	1.16E-06	0.56 ± 0.10	**3.17E-08**
cg04276536^e^	16	57567813	*CCDC102A*	-0.85 ± 0.15	**1.96E-08**	-0.45 ± 0.21	2.99E-02	-0.58 ± 0.18	1.24E-03	-0.64 ± 0.17	1.31E-04
cg24069602	17	62045522	*SCN4A*	0.47 ± 0.14	1.19E-03	0.57 ± 0.17	1.09E-03	0.46 ± 0.09	6.97E-07	0.51 ± 0.09	**3.39E-08**

^a^Annotated manually from the UCSC genome browser either nearest gene within 50 kb.

^b^Random effects and standard errors have been multiplied by 100 and therefore represent percentage differences in methylation between cases and controls.

^c^or to alternative transcript of refseq gene.

^d^CpG has known cis-mQTL with effects towards hypomethylation.

^e^CpG has known cis-mQTL with effects towards hypermethylation.

^f^CpG – cytosine-(phosphate)-guanine site; S.E – standard error; N.B. *P*-values less than 2.4 × 10e-07 are in bold and those less than 3.6 × 10e-08 are shaded.

We identified a larger number of DMPs associated with schizophrenia using the sex-stratified model compared to the sex-adjusted model. This pattern remained similar when a more conservative *p*-value threshold was applied with only one DMP identified in the sex-adjusted and 10 DMPs in the sex-stratified analysis (Table 2 and Supplementary Information Figs. S4 & S5). All DMPs identified in the sex-adjusted model except cg12269535 (*SRF*) were also identified using the sex-stratified model. Notably, all DMPs identified in these two models had concordant effect directions in males and females (Table 2). For the total sample, half (99) of the top 200 DMPs in the sex-stratified and sex-adjusted models were identical. In the sex-specific analyses, two of the top 200 DMPs were common to the male-only and female-only analyses further supporting sex differences in DNA methylation changes associated with schizophrenia (Supplementary Tables S2–S5; Supplementary Information Fig. S6).

In the sex-specific analyses, we identified one and four DMRs associated with schizophrenia in males and females, respectively. We found 20 DMRs associated with schizophrenia in the sex-stratified analysis of the total sample. Two of the DMRs identified in females (chr11: 76380921–76381166, chr17: 7832764–7832944), and the DMR identified in males (chr10: 104535792–104536036) were also among those identified in the total sample. As expected, most of the DMRs associated with schizophrenia harbored at least one protein-coding gene (Table 3).

**Table 3:** Differentially methylated regions associated with schizophrenia in sex-specific and sex-stratified meta-analyses.

Population/Method	CHR	Start BP	End BP	Probes	Genes	*P*-value	Sidak *P*-value
Female	6	31545252	31545474	5	*TNF*	1.35E-08	2.87E-05
	11	76380921	76381166	6	*LRRC32*	5.16E-09	9.92E-06
	12	122018897	122019118	6	*KDM2B*	8.24E-10	1.76E-06
	17	7832764	7832944	6	*KCNAB3*	6.65E-09	1.74E-05
Male	10	104535792	104536036	7	*WBP1L*	4.32E-11	8.35E-08
Total sample: sex-stratified	1	57110867	57111200	8	*PRKAA2*	6.74E-11	9.54E-08
	1	91852791	91853090	6	*HFM1*	8.03E-10	1.27E-06
	1	32827707	32827841	6	*FAM229A; TSSK3*	6.76E-09	2.38E-05
	3	24536231	24537051	11	*THRB*	4.56E-13	2.62E-10
	3	158390329	158390526	5	*LXN; GFM1*	2.12E-09	5.06E-06
	3	99594931	99595146	4	*CMSS1; FILIP1L*	2.14E-08	4.70E-05
	5	135364328	135364581	8	*TGFBI*	2.78E-08	5.18E-05
	5	71146767	71146877	6	*CARTPT*	5.01E-09	2.15E-05
	6	29601398	29601557	5	*GABBR1*	2.65E-07	7.84E-04
	7	49813031	49813112	6	*VWC2*	6.32E-08	3.67E-04
	7	142494148	142494245	4	*TRBC2*	2.78E-09	1.35E-05
	10	104535792	104536122	9	*WBP1L*	9.18E-12	1.31E-08
	11	111847968	111848401	7	*DIXDC1*	5.39E-15	5.92E-12
	11	76380921	76381166	6	*LRRC32*	5.97E-08	1.15E-04
	17	7832764	7833238	8	*KCNAB3*	3.30E-13	3.28E-10
	19	58095424	58095660	8	*ZIK1*	3.22E-07	6.43E-04
	19	54876446	54876796	7	*LAIR1*	2.41E-10	3.24E-07
	19	8274853	8275230	4	*CERS4*	1.22E-09	1.53E-06
	20	36148642	36149023	17	*BLCAP; NNAT*	8.30E-07	1.03E-03
	22	19949873	19950041	6	*COMT*	2.35E-07	6.59E-04

*CHR* chromosome, *BP* base position based on the Human genome build 19.

### Regulatory enrichment and functional analyses

Known *cis*-meQTL were identified for seven of the DMPs associated with schizophrenia in sex-specific and sex-stratified analyses. The genetic effects were towards hypomethylation on two of these DMPs, and hypermethylation on the remaining five. Interestingly, only two DMPs (cg27207470, and cg01678084) demonstrated concordant effect directions of known *cis*-meQTLs in blood and the association we found for schizophrenia (Table 2). The genetic effects for these two DMPs were reported in adolescence, at birth or in childhood (Supplementary Tables S6–S10). In gene set analyses for the genes annotated to the DMRs identified in the sex-stratified analyses, no enrichment for biological processes or cellular components were significant after correction for multiple testing.

### Simulation of EWAS with a stronger effect of methylation in females versus males

The simulations were set up based on the following observations. First, our sex-stratified analysis led to identification of a larger number of DMPs than the sex-adjusted one. Second, a recent study identified a greater burden of DNA methylation change in the brain of female than male patients with schizophrenia [17]. Therefore, we compared sex-adjusted and sex-stratified models in four scenarios that differed by the degree of effect differences in males and females.

**Fig. 2:**
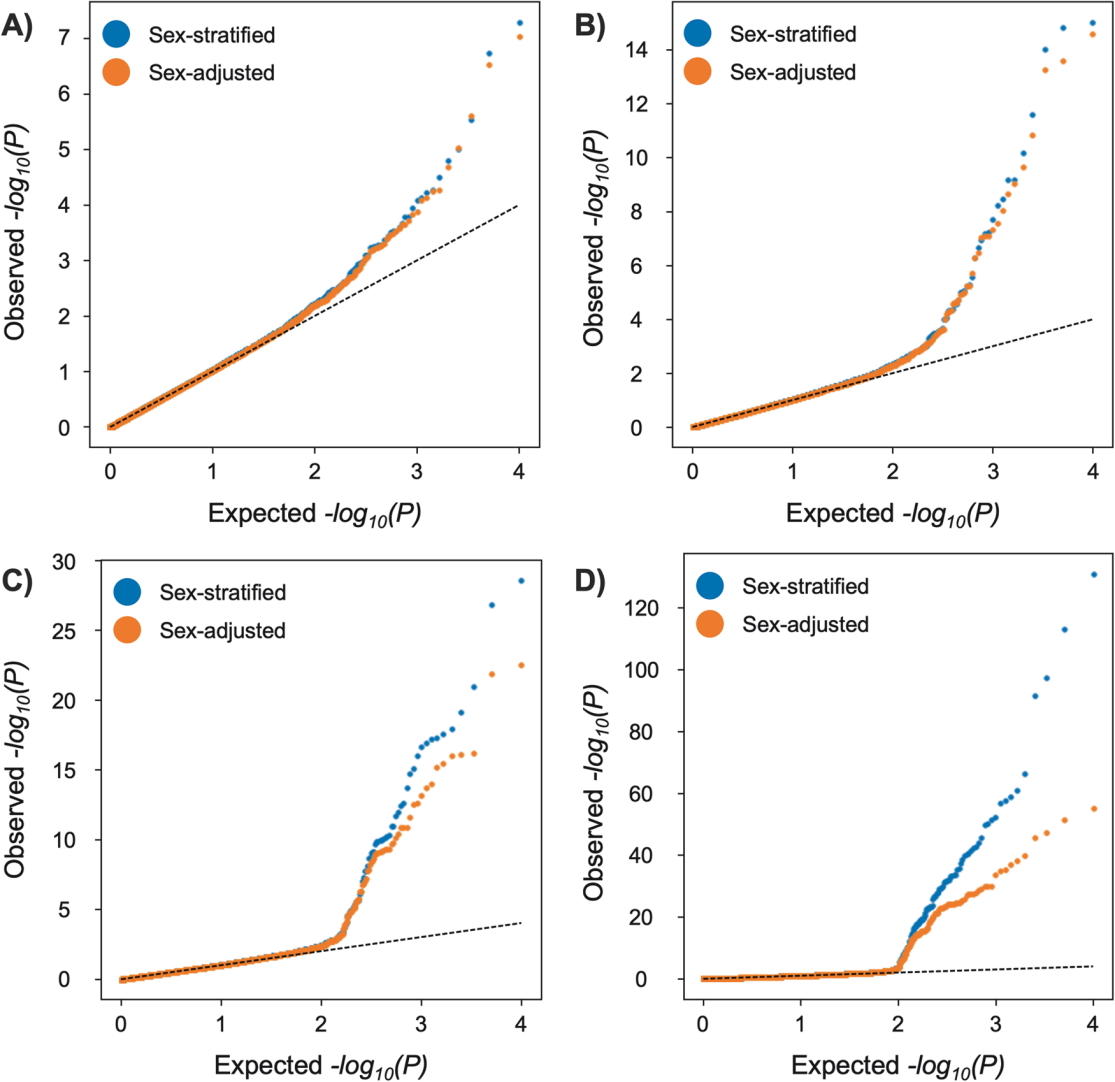
QQ plot comparison of sex-adjusted and sex-stratified meta-analyses models in a simulation set-up. Scenario **A** effect size in females = males, scenario **B** effect size in females= twice that of males, scenario **C** effect size in females = four times that of males, and scenario **D** effect size in females = eight times that of males.

The QQ plots in the simulation demonstrated greater leftward and upward deviation (and thus greater statistical power) in the sex-stratified analysis compared to the sex-adjusted analysis. The advantage of sex-stratified analysis increased proportionally to the difference between average effect sizes in males and females (Fig. 2A–D). Similarly, sex-stratified analyses in our data from the cohorts had QQ plots that exhibited greater leftward and upward deviation compared to sex-adjusted analysis (Supplementary Information Fig. S7 A, B).

### Validation: polymethylation scores as predictors of schizophrenia

To verify whether the improved power observed from a sex-stratified analysis translates into improved schizophrenia prediction, we calculated PMS using four meta-analyses (sex-stratified, sex-adjusted, male-only, female-only) of UCL, IoPPN, and ABR cohorts as training datasets and tested the performance of the PMSs in the TOP total sample, TOP male-only, and TOP female-only datasets.

**Fig. 3:**
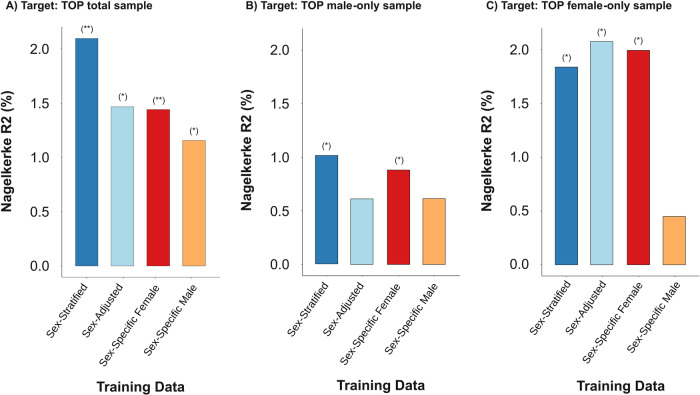
The variance in schizophrenia liability explained by polymethylation scores (PMS). The variance in liability is depicted by the Nagelkerke R^2^ (y-axis), derived from a logistic regression model. PMS derived from sex-stratified (dark blue bar), sex-adjusted (light blue bar), sex-specific female (red bar), and sex-specific male (orange bar), and their performance was tested on **A** total, **B** male-only, and **C** female-only samples. PMS associated with schizophrenia at *p*-value < 0.001 (**) and *p*-value < 0.05 (*).

As shown in Fig. 3A, all four PMSs were associated with schizophrenia with the TOP total sample as the target data. The PMS derived from the sex-stratified analysis (Nagelkerke R^2^ = 0.021, *p*-value = 1.63 × 10^-5^) yielded the highest prediction for schizophrenia with the lowest AIC and BIC values (Supplementary Information Table S11). Notably, the prediction of schizophrenia by the PMS from the sex-adjusted analysis (Nagelkerke R^2^ = 0.015, *p*-value = 0.0003) was smaller than that from the sex-stratified analysis (*p* = 0.036). Interestingly, the variance explained by the PMS from sex-specific female (Nagelkerke R^2^ = 0.014) and male (Nagelkerke R^2^ = 0.012) analyses were not different from that from the sex-adjusted analysis (*p* > 0.05).

Interestingly, only the PMSs from the sex-stratified (Nagelkerke R^2^ = 0.010, *p*-value = 0.025) and from the sex-specific female analyses (Nagelkerke R^2^ = 0.009, *p*-value = 0.036) predicted schizophrenia in the male target data (Fig. 3B). In the female sample as target data, the PMSs from the sex-adjusted (Nagelkerke R^2^ = 0.021, *p*-value = 0.004), the sex-specific female (Nagelkerke R^2^ = 0.020, *p*-value = 0.005) and the sex-stratified analyses (Nagelkerke R^2^ = 0.018, *p*-value = 0.007) predicted schizophrenia to a similar degree (Fig. 3C). Based on AIC and BIC values, the PMS derived from the sex-stratified analysis provided the best model fit in the prediction of schizophrenia in the total and male-only target data, while the PMS from the sex-adjusted model offered the best model fit in the female-only target data (Supplementary Information Table S11).

## Discussion

In the current study, our systematic comparisons of schizophrenia EWAS meta-analyses and simulations revealed that a sex-stratified approach improves the power as evidenced by consistent upward and leftward deviations in the QQ plots. Consequently, the sex-stratified meta-analysis resulted in the identification of a larger number of DMPs associated with schizophrenia than the standard sex-adjusted approach. Our simulations also indicate that the gain in power from sex-stratified analysis is more pronounced when there are substantial differences in sex-specific effect estimates. This translated into a stronger association of schizophrenia status with PMS derived from the sex-stratified results than PMS from the sex-adjusted model. Interestingly, PMS associations with schizophrenia tended to be stronger in females than in males regardless of the training dataset. The boost in the power obtained from the sex-stratified EWAS analytical approach shows the methodological advantage and expands our understanding of the molecular pathways involved in the pathophysiology of schizophrenia.

Our application of a sex-stratified analytical approach boosted the discovery of DNA methylation loci. Although the advantages of using sex-stratification in GWAS meta-analyses have previously been suggested [18], our study is the first to systematically examine the method in the EWAS of schizophrenia. The sex-stratified analysis improved the discovery of DMPs with concordant effect direction in both sexes but with different magnitudes [18]. This observation is in agreement with the recent report from Zhou et al. who observed a larger magnitude of changes in DNA methylation associated with schizophrenia in females than in males [17]. Thus, further supporting the rationale for a sex-stratified meta-analysis approach in EWAS of schizophrenia. However, neither sex-stratified nor sex-adjusted meta-analyses adequately capture DNA methylation changes with discordant directions in males and females as the effect estimates may cancel each other out [17].

The identification of DMRs unique to females in the sex-specific approach suggests that some molecular pathways involved in the pathophysiology of schizophrenia might be sex dependent. Studies have shown more changes in DNA methylation and gene expression associated with schizophrenia in females than in males in postmortem prefrontal cortex [16, 17], and in neuronal cell lines from schizophrenia-discordant monozygotic twins [44]. These sex differences may also relate to the sex-specific effects of environmental factors on the epigenome [25], or sex-specific resilience [17]. The existence of sex differences in the biology of schizophrenia was previously reported, however, the final molecular pathways leading to the symptoms may be similar [44]. Furthermore, DNA methylation changes with large effect sizes in females are more likely to be identified [17], which may account for some of these sex differences.

The genes mapped to the identified DMRs link to genetic, biological, and environmental factors relevant to understanding the pathophysiology of schizophrenia. GWAS of schizophrenia has identified a locus in the gene *GABBR1* in East Asian population [45]. *GABBR1* encodes the receptor for the main inhibitory neurotransmitter, gamma-aminobutyric acid (GABA), in the brain [46]. Also, candidate gene studies have reported associations between schizophrenia and *THRB* [47], *COMT* [48], *DIXDC1* [49], and *WBP1L* [50]. Other genes *LRRC32*, *KCNAB3*, *NNAT*, *TGFB1*, *VWC2*, and *KDM2B* were linked to neurodevelopmental processes and neuropsychiatric phenotypes [51–56]. Additionally, differential methylation involving *LRRC32* and *BLCAP*/*NNAT* were previously linked to exposure to adverse perinatal factors (e.g., viral infection, iron deficiency, birth asphyxia) with potential neuropsychiatric consequences [25, 51]. Furthermore, some of the genes mapped to the DMRs are involved in molecular pathways relevant to the immune system (*LAIR1*, *TNF*) [57, 58], regulation of gene expression (*KDM2B*, *ZIK1*) [59, 60], and energy metabolism (*PRKAA2*) [61]. The identification of these genes sheds light on the pathways involved in the pathophysiology of schizophrenia [62].

DNA methylation changes in *COMT* have not been reported in previous EWAS of schizophrenia. *COMT* encodes the enzyme which plays a vital role in the methylation of catecholamines (e.g., dopamine) and is a drug target for neuropsychiatric disorders [63]. The identification of a novel DMR associated with schizophrenia mapping to *KCNAB3*, a gene coding subunit of voltage-gated potassium ion channel [64], may also have the potential as a drug target. The gene and its product are linked to migraine and epilepsy [52, 65]. A recent animal study reported that KCNAB2, a product of a gene closely related to *KCNAB3*, is involved in the regulation of the firing of dopaminergic neurons [66]. Dysregulation of dopaminergic neurotransmission has been suggested as a final common pathway of multiple genetic and environmental factors known to be risk factors for schizophrenia [67]. DNA methylation changes at *COMT* and *KCNAB3* may be one of the pathways involved in the pathophysiology of schizophrenia at least in a subgroup of patients although this needs further research.

To progress towards the use of EWAS results in predicting schizophrenia, we have tested the potential for the different models to identify sets of methylation signals identified in a discovery sample, which could predict disease status in an independent sample. Not surprisingly, since sex-stratified analyses capture better effects that differ between sexes, PMS prediction using sex-stratified analysis performed better than sex-adjusted PMS and might thus be more efficient than the standard approach. Moreover, the observation that the sex-adjusted PMS yields the highest Nagelkerke R^2^ values when applied to the female sample but does not explain variability in schizophrenia in the male sample suggests that this approach may be significantly influenced by the greater magnitude of DNA methylation changes in females. Accordingly, epigenetic discoveries in schizophrenia using sex-adjusted models may lead to sex disparities. The significant association between PMS derived from the sex-specific female but not that from sex-specific male with schizophrenia status in the male sample was unexpected and needs further investigation. Based on the reported greater DNA methylation dysregulation in females with schizophrenia [17], we speculate the sex-specific female EWAS may be more powerful than sex-specific male ones for a given sample size. Consequently, the PMS derived from sex-specific female analysis appears to perform better in predicting schizophrenia across all target datasets compared to the PMS from sex-specific male analysis.

We acknowledge that our study has important limitations. The DNA methylation probes provide coverage of a small fraction of the human epigenome and therefore, it does not capture effects for the regions of the genome that are not covered. Furthermore, the DNA methylation changes in the blood are not identical to those in the brain, however, there is a significant similarity between the two [5, 68]. DNA methylation changes may partly be a result of treatment [20] or other consequences of schizophrenia, and the PMS can be conceived as an ‘epigenetic classifier’ rather than a mere predictor of ‘risk’ for disease. The cross-sectional study designs of the cohorts included in our analyses do not allow causal associations to be established. Although the identified DNA methylation changes need further investigation for their functional molecular effects, our findings are still relevant for the development of biomarkers of schizophrenia especially since blood is an accessible tissue. We presented the identified DMPs, DMRs, and associated biological pathways after applying corrections for multiple tests in each of the approaches. While presenting all these findings could arguably lead to an increase in false positives, it is important to note that these approaches are complementary, providing a more comprehensive interpretation of the results.

In conclusion, we have shown that sex differences in the epigenome and sex-stratified analysis can be leveraged to enhance epigenetic discoveries in schizophrenia. These epigenetic discoveries help advance our understanding of the molecular pathways involved in the pathophysiology of schizophrenia. The advantages of sex-stratified analysis extend beyond the discovery of novel loci to improved prediction using PMS, which may have potential clinical applications. The potential downstream effects of DNA methylation changes warrant further research to advance precision psychiatry. Future investigations with larger coverage of the epigenome are needed to reveal the male and female differences contributing to the phenotypic heterogeneity. Given that a significant proportion of DMPs associated with schizophrenia are under the influence of genetic variants [5, 17], the advantage of sex-stratified analysis may also extend to genetic studies highlighted previously [18]. We show that sex differences have a potential implication for the development of sex-specific biomarkers, enhance prediction scores, improve our understanding of the pathophysiology and thus perhaps progress towards precision psychiatry.

## Supplementary information

Supplementary Information

Supplementary Tables S1-S5

Supplementary Tables S6-S10

## Data Availability

DNA methylation and phenotype data for UCL, IoPPN, and ABR cohorts can be freely downloaded from the Gene Expression Omnibus at https://www.ncbi.nlm.nih.gov/geo/, GEO accession numbers: GSE80417, GSE152027, and GSE84727 respectively. EWAS summary statistics for the TOP cohort will be available from Professor Stephanie Le Hellard, University of Bergen.

## Code availability

Code used for these analyses can be obtained freely from the corresponding authors upon request.
