# Supplementary material for: Sex effects on DNA methylation affect discovery in epigenome-wide association study of schizophrenia
Source: Mol Psychiatry. 2024 Mar 19;29(8):2467–77. doi: 10.1038/s41380-024-02513-9 (PMC11412896; doi:10.1038/s41380-024-02513-9)
Supplement: Supplementary file 1 — Supplementary Information [file 41380_2024_2513_MOESM1_ESM.docx]

Supplementary Information for:

Sex effects on DNA methylation affect discovery in epigenome-wide association study of schizophrenia.

Markos Tesfaye, MD, PhD,^‡^ ^1, 2^ Leticia M. Spindola, PhD,^‡^ ^2, 3, 4^ Anne-Kristin Stavrum, PhD,^2, 3^ Alexey Shadrin, PhD,^1, 5^ Ingrid Melle, MD, PhD,^1^ Ole A. Andreassen, MD, PhD,^1, 5^ and Stephanie Le Hellard, PhD ^2, 3, 4^

^1^ NORMENT, Division of Mental Health and Addiction, Oslo University Hospital and Institute of Clinical Medicine, University of Oslo, Oslo, Norway

^2^ NORMENT, Department of Clinical Science, University of Bergen, Bergen, Norway

^3^ Dr. Einar Martens Research Group for Biological Psychiatry, Department of Medical Genetics, Haukeland University Hospital, Bergen, Norway.

^4^ Bergen Center for Brain Plasticity, Haukeland University Hospital, Bergen, Norway.

^5^ KG Jebsen Centre for Neurodevelopmental Disorders, University of Oslo and Oslo University Hospital, Oslo, Norway

**^‡^ Contributed equally**

**Page 2** **Figure S1:** Forest plots showing the random effects model for a differentially methylated position (cg27541604) association with schizophrenia in sex-specific female (A), sex-specific male (B), sex-adjusted (C), and sex-stratified (D) meta-analyses. SMD: standardized mean differences between cases and controls; SE: standard error; CI: confidence interval.

**Page 3 Figure S2:** Manhattan plot of DNA methylation loci association with schizophrenia in a meta-analysis of male-only samples.

**Page 4 Figure S3:** Manhattan plot of DNA methylation loci association with schizophrenia in a meta-analysis of female-only samples.

**Page 5 Figure S4:** Manhattan plot of DNA methylation loci association with schizophrenia in sex-stratified meta-analysis.

**Page 6 Figure S5:** Manhattan plot of DNA methylation loci association with schizophrenia in sex-adjusted meta-analysis.

**Page 7 Figure S6:** Venn diagram showing the number of overlapping top 200 differentially methylated probes in sex-specific female, sex-specific male, sex-stratified, and sex-adjusted meta-analyses.

**Page 8 Figure S7**: QQ plots of sex-stratified and sex-adjusted meta-analyses of EWAS of schizophrenia. A – ABR, UCL, IoPPN, and TOP cohorts. B – ABR, UCL, and IoPPN cohorts.

**Page 9 Table S11:** Polymethylation score prediction model comparison metrics and statistics. Models were compared within target datasets.





**Figure S1:** Forest plots showing the random effects model for a differentially methylated position (cg27541604) association with schizophrenia in sex-specific female (A), sex-specific male (B), sex-adjusted (C), and sex-stratified (D) meta-analyses. SMD: standardized mean differences between cases and controls; SE: standard error; CI: confidence interval.


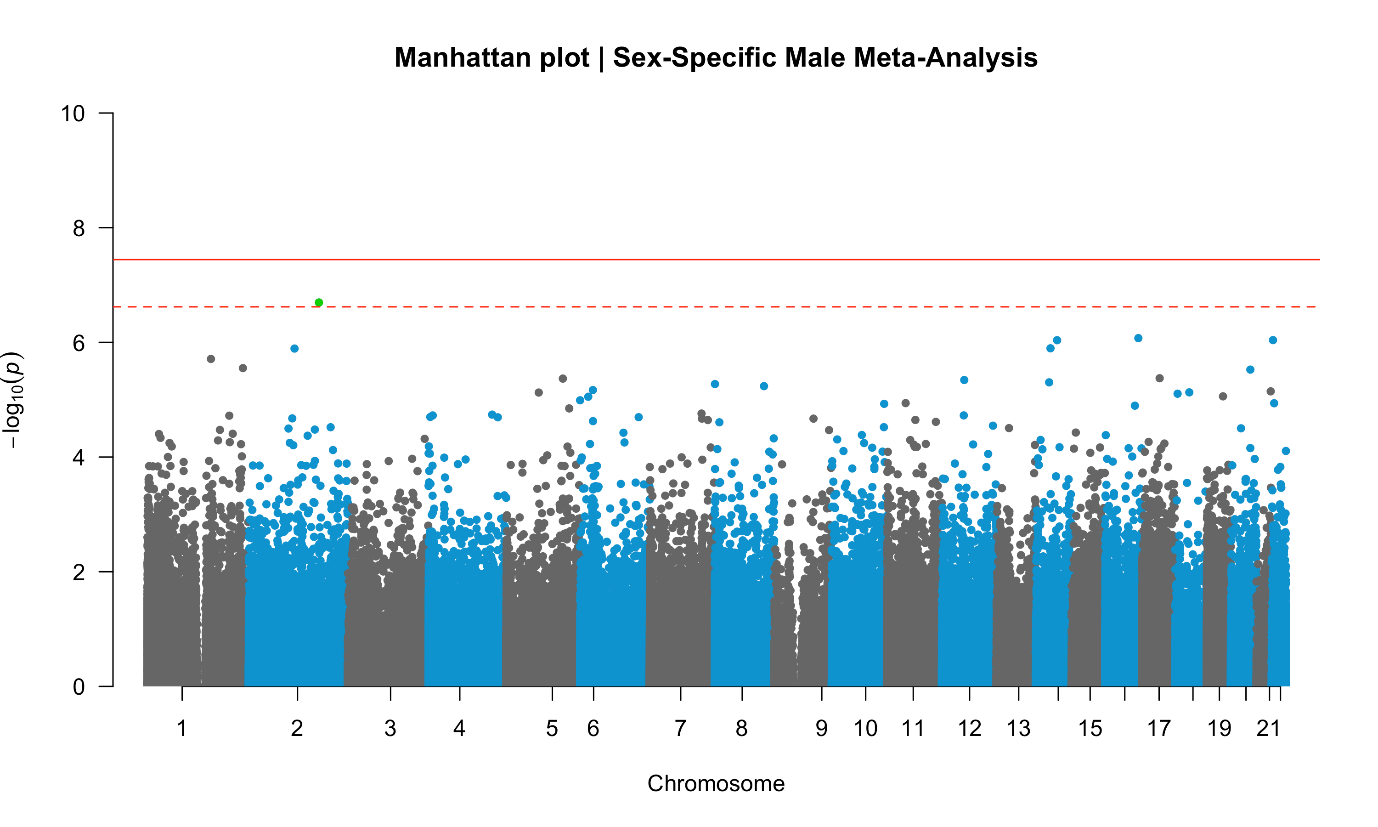


**Figure S2:** Manhattan plot of DNA methylation loci association with schizophrenia in a meta-analysis of male-only samples. The continuous red line represents the genome-wide significance threshold (3.6 × 10E-08), and the dashed red line represents the 450K threshold (2.4 × 10E-07). Loci associated with schizophrenia with a p-value less than 2.4 × 10E-07 threshold are highlighted in green.


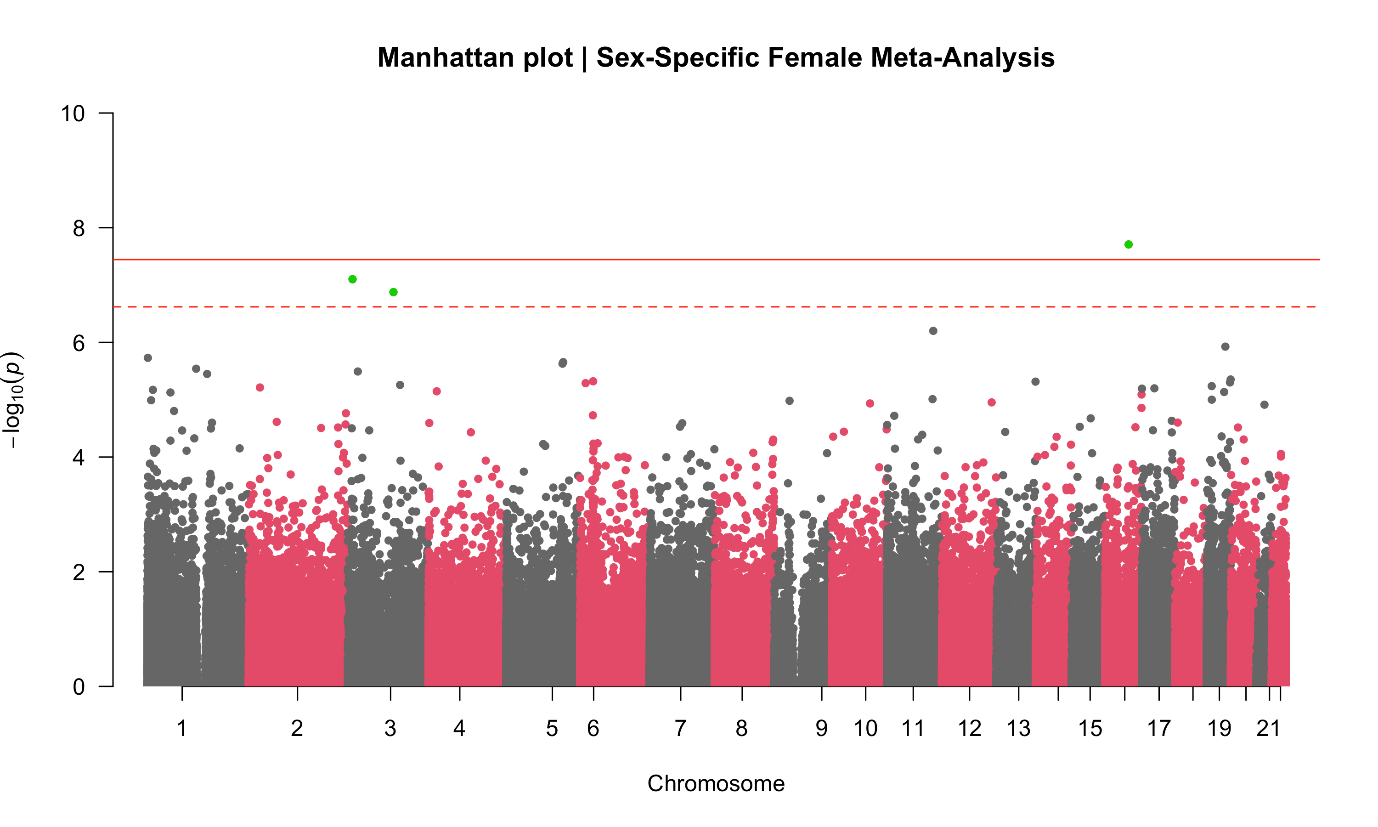


**Figure S3:** Manhattan plot of DNA methylation loci association with schizophrenia in a meta-analysis of female-only samples. The continuous red line represents the genome-wide significance threshold (3.6 × 10E-08), and the dashed red line represents the 450K threshold (2.4 × 10E-07). Loci associated with schizophrenia with a p-value less than 2.4 × 10E-07 threshold are highlighted in green.


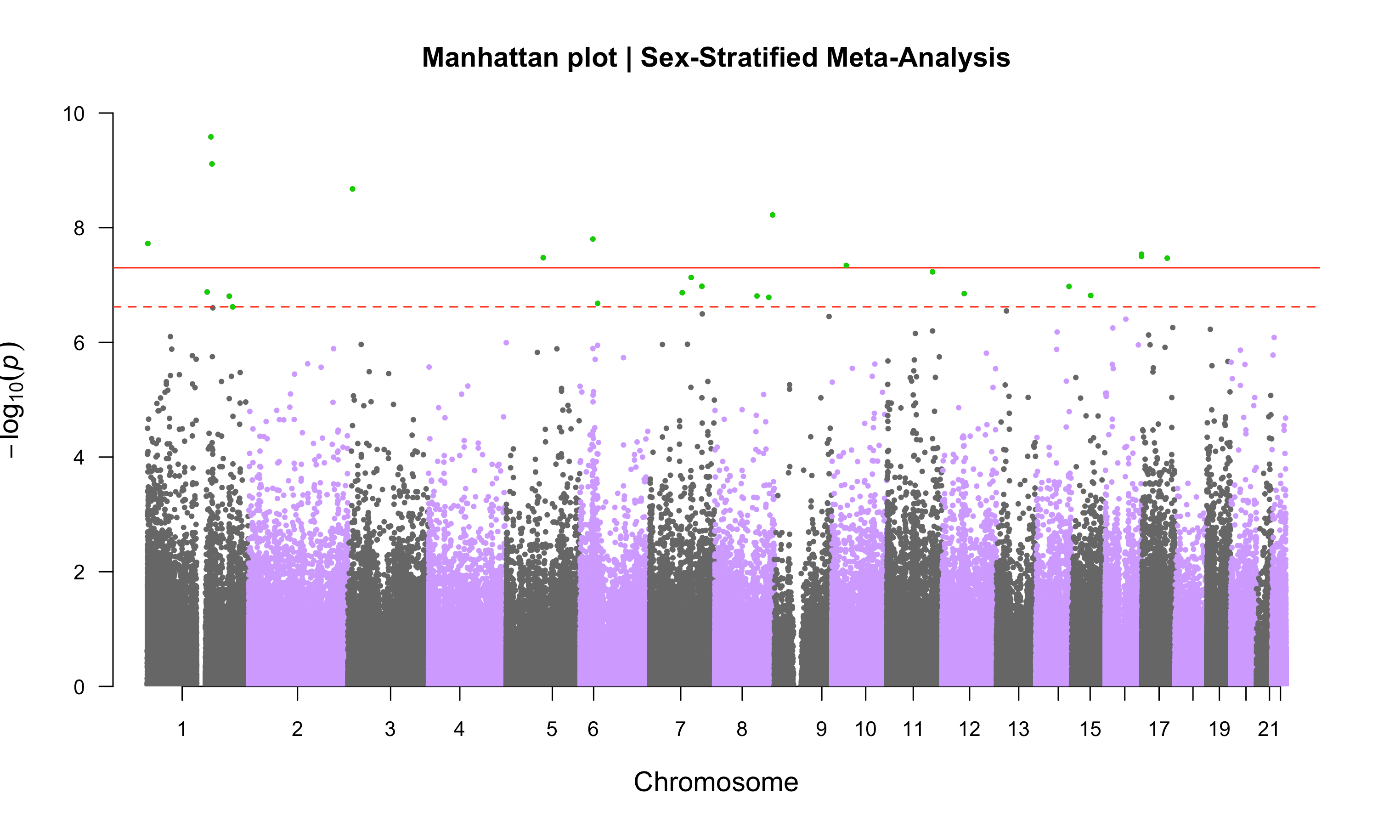


**Figure S4:** Manhattan plot of DNA methylation loci association with schizophrenia in sex-stratified meta-analysis. The continuous red line represents the genome-wide significance threshold (3.6 × 10E-08), and the dashed red line represents the 450K threshold (2.4 × 10E-07). Loci associated with schizophrenia with a p-value less than 2.4 × 10E-07 threshold are highlighted in green.


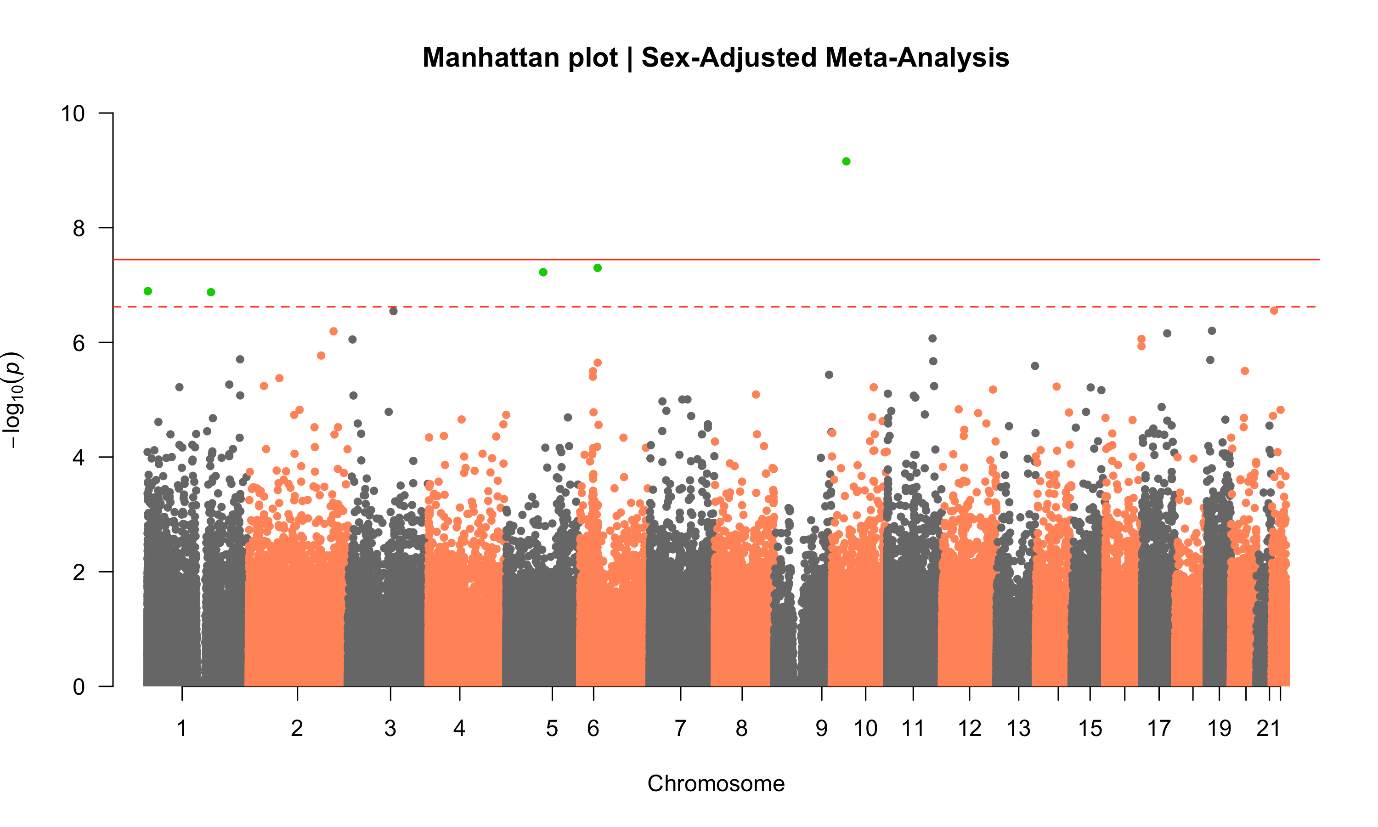


**Figure S5:** Manhattan plot of DNA methylation loci association with schizophrenia in sex-adjusted meta-analysis. The continuous red line represents the genome-wide significance threshold (3.6 × 10E-08), and the dashed red line represents the 450K threshold (2.4 × 10E-07). Loci associated with schizophrenia with a p-value less than 2.4 × 10E-07 threshold are highlighted in green.


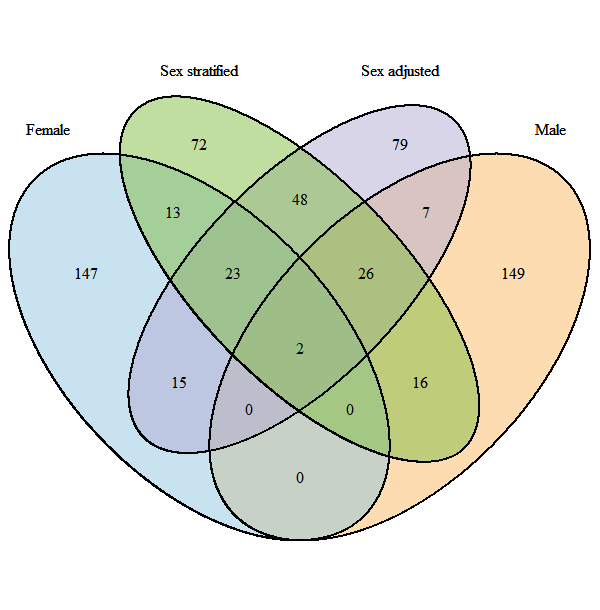


**Figure S6:** Venn diagram showing the number of overlapping top 200 differentially methylated probes in sex-specific female, sex-specific male, sex-stratified, and sex-adjusted meta-analyses.


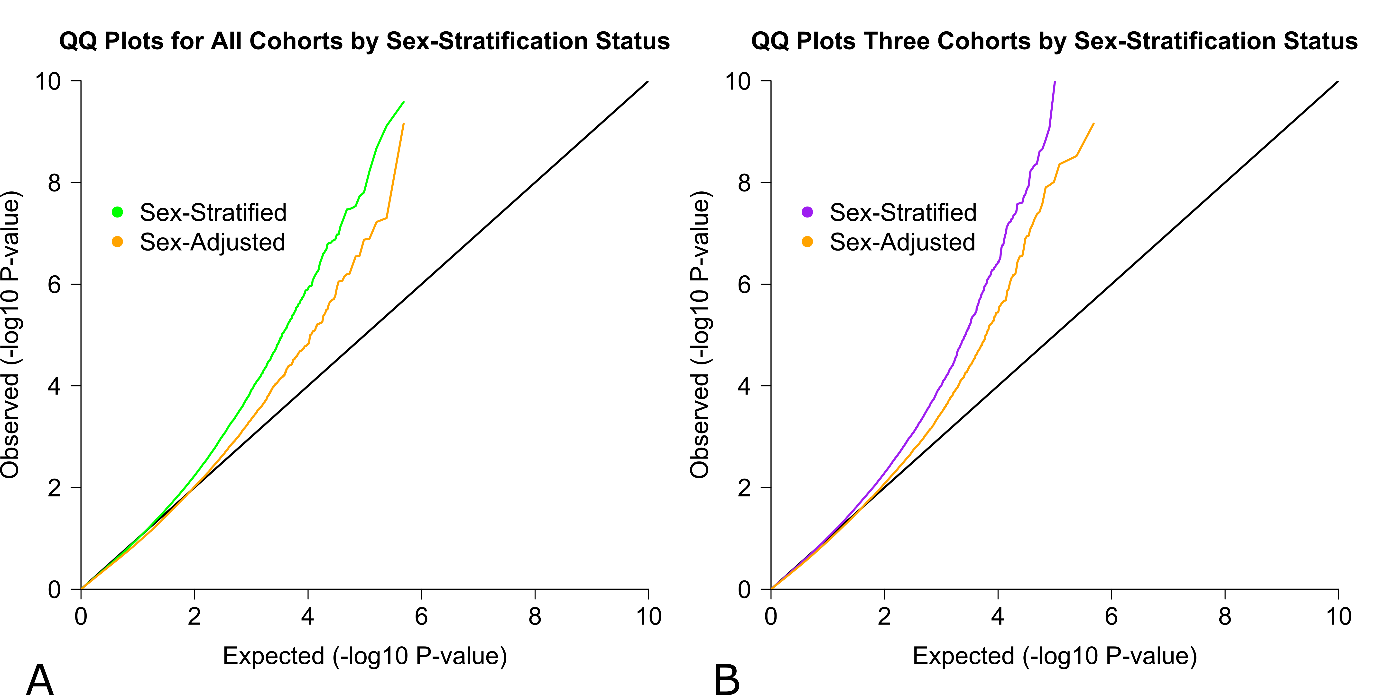


**Figure S7**: QQ plots of sex-stratified and sex-adjusted meta-analyses of EWAS of schizophrenia. A – ABR, UCL, IoPPN, and TOP cohorts. B – ABR, UCL, and IoPPN cohorts.

| **Table S11:** Polymethylation score prediction model comparison metrics and statistics. Models were compared within target datasets. | | | |
| --- | --- | --- | --- |
| Target dataset | Training dataset | AIC | BIC |
| TOP total sample | **Sex-stratified** | **1610.35** | **1620.57** |
|  | Sex-adjusted | 1616.09 | 1626.32 |
|  | Sex-specific female | 1616.33 | 1626.55 |
|  | Sex-specific male | 1618.92 | 1629.14 |
| TOP male-only | **Sex-stratified** | **904.53** | **913.58** |
|  | Sex-adjusted | 906.56 | 915.61 |
|  | Sex-specific female | 905.19 | 914.24 |
|  | Sex-specific male | 906.54 | 915.59 |
| TOP female-only | Sex-stratified | 716.29 | 724.89 |
|  | **Sex-adjusted** | **715.33** | **723.93** |
|  | Sex-specific female | 715.67 | 724.27 |
|  | Sex-specific male | 721.88 | 730.48 |
|  | Model comparison | *z-statistics* | p-value |
| TOP total sample | Sex-stratified *vs.* Sex-adjusted | 1.801 | 0.036 |
|  | Sex-stratified *vs.* Sex-specific female | 0.954 | 0.170 |
|  | Sex-stratified *vs.* Sex-specific male | 1.495 | 0.067 |
|  | Sex-adjusted *vs.* Sex-specific female | 0.039 | 0.484 |
|  | Sex-adjusted *vs.* Sex-specific male | 0.577 | 0.282 |
|  | Sex-specific female *vs.* Sex-specific male | 0.336 | 0.368 |
| TOP male-only | Sex-stratified *vs.* Sex-adjusted | 1.123 | 0.131 |
|  | Sex-stratified *vs.* Sex-specific female | 0.210 | 0.417 |
|  | Sex-stratified *vs.* Sex-specific male | 0.662 | 0.254 |
|  | Sex-adjusted *vs.* Sex-specific female | -0.428 | 0.334 |
|  | Sex-adjusted *vs.* Sex-specific male | -0.009 | 0.496 |
|  | Sex-specific female vs. Sex-specific male | 0.322 | 0.374 |
| TOP female-only | Sex-stratified *vs.* Sex-adjusted | -0.425 | 0.335 |
|  | Sex-stratified *vs.* Sex-specific female | -0.138 | 0.445 |
|  | Sex-stratified *vs.* Sex-specific male | 1.440 | 0.075 |
|  | Sex-adjusted *vs.* Sex-specific female | 0.067 | 0.473 |
|  | Sex-adjusted *vs.* Sex-specific male | 1.551 | 0.060 |
|  | Sex-specific female *vs.* Sex-specific male | 1.118 | 0.132 |
| AIC: Akaike Information Criterion; BIC: Bayesian Information Criterion; z-statistics and p-values derived from Vuong non-nested hypothesis test. The lowest AIC and BIC values are highlighted in bold. | | | |
